# Modular Pressure and Flow Rate-Balanced Microfluidic Serial Dilution Networks for Miniaturised Point-of-Care Diagnostic Platforms

**DOI:** 10.3390/s19040911

**Published:** 2019-02-21

**Authors:** Nikolaos Vasilakis, Konstantinos I. Papadimitriou, Hywel Morgan, Themistoklis Prodromakis

**Affiliations:** 1 Nanoelectronics & Nanotechnology Research Group, Electronics and Computer Science, University of Southampton, Southampton SO17 1BJ, UK; k.papadimitriou@ucl.ac.uk (K.I.P.); hm@ecs.soton.ac.uk (H.M.); 2 Institute for Life Sciences, University of Southampton, Southampton SO17 1BJ, UK; 3 Zepler Institute for Photonics and Nanoelectronics, University of Southampton, Southampton SO17 1BJ, UK; t.prodromakis@soton.ac.uk

**Keywords:** serial diluter, pressure balance, flow rate balance, Lab-on-PCB, Microfluidics, Point-of-Care diagnostics, PMMA microfluidics, PCB manufactured microfluidics, step wise diluter, modular serial diluter

## Abstract

Fast, efficient and more importantly accurate serial dilution is a necessary requirement for most biochemical microfluidic-based quantitative diagnostic applications. Over the last two decades, a multitude of microfluidic devices has been proposed, each one demonstrating either a different type of dilution technique or complex system architecture based on various flow source and valving combinations. In this work, a novel serial dilution network architecture is demonstrated, implemented on two entirely different substrates for validation and performance characterisation. The single layer, stepwise serial diluter comprises an optimised microfluidic network, where identical dilution ratios per stage are ensured, either by applying equal pressure or equal flow rates at both inlets. The advantages of this serial diluter are twofold: Firstly, it is structured as a modular unit cell, simplifying the required fluid driving mechanism to a single source for both sample and buffer solution. Thus, this unit cell can be used as a fundamental microfluidic building block, forming multistage serial dilution cascades, once combined appropriately with itself or other similar unit cells. Secondly, the serial diluter can tolerate the inevitable flow source fluctuations, ensuring constant dilution ratios without the need to employ damping mechanisms, making it ideal for Point of Care (PoC) platforms. Proof-of-concept experiments with glucose have demonstrated good agreement between simulations and measurements, highlighting the validity of our serial diluter.

## 1. Introduction

Almost every quantitative chemical and biological assay relies heavily upon accurate and prompt serial dilution. Its usefulness lies in the fact that with a suitable sample preparation procedure, a wide variety of known scaled concentration samples can be generated using a suitable diluent [1]. For example, widely known quantitative assays, such as real time polymerase chain reaction (q-PCR) or antigen assays, e.g., Enzyme-Linked Immunosorbent Assay (ELISA), require serial dilutions of a known analyte to produce several reference calibration signals which can be later used for standard or calibration curves. Over the last 15 years, many continuous-flow microfluidic dilution devices have been proposed for the generation of multiple stepwise concentrations or concentration gradients with logarithmic [2,3] or linear [4,5,6,7] dilution. However, the majority often requires multiple flow sources (pressure or flow rate) to be employed for driving fluids through the device. Their operation could be potentially unstable and they may be hard to handle [8] and integrate with other components in a monolithic manner.

There are two main categories of diluters; (a) diffusion-based and (b) multi-step diluters. The diffusion-based designs [9,10] exploit the concentration gradient formed along a microfluidic channel to create samples of different concentrations across the same microchannel. The main challenge for these devices is to obtain linear concentration gradients, due to the inherent non-linear diffusion mechanism [11]. Multi-step microfluidic dilution devices were firstly proposed by Jacobson et al. in 1999 but the resulting dilution ratio was neither linear nor logarithmic [12]. In 2001 the Whitesides’ group proposed the first linear concentration gradient device [13], while two years later an improved device following the same approach, including staggered herringbone micromixers, was demonstrated [14]. Kim et al. developed a serial dilution microfluidic chip that produces logarithmic and linear step-wise concentrations, respectively. However, the inlet flow rates of both buffer and sample were not equal. The logarithmic device required a buffer-to-sample ratio of 1:36 (sample 0.5 mL/h vs buffer 18.0 mL/h) while the linear one needed a 1:2.75 ratio (sample 1.0 mL/h vs buffer 2.75 mL/h) [11]. Therefore, this device is not very practical for a PoC implementation, since it can only operate with syringe pumps, which will inevitably introduce pressure fluctuations up to 50% [15,16]. In PoC applications, these variations cannot be mitigated by employing damping mechanisms (e.g., long elastic tubing) due to space limitations. In fact, these variations will be enhanced because of the cost restrictions of the handheld device that will not allow for a sophisticated syringe pump mechanism to be implemented. Hence, flow oscillations from two independent syringe pumps, driving sample and diluent through the device, will practically neither have the same initial phase nor the same oscillating frequency (different leadscrew angular velocity). Therefore, the stability of the dilution ratio will be strongly affected, which will subsequently affect the overall system performance [15,16]. Lee et al. developed a microfluidic network-based device consisting of three different layers. This design could combine three different samples with a buffer solution. The buffer solution diluted the samples in a ratio 1:4 and through the 3-layer microfluidic network all possible combinations were formed in the output device ports. Nonetheless, the buffer flow rate was four times higher the flow rate of each sample [17]. Recently, this principle was enhanced by adjusting not only the channel length but also the channel width. In 2013, Weibull et al. reported a stepwise dilution generator, including logarithmic and linear gradients in a two-layer microfluidic implementation. Although two different dilutions gradients were implemented on the same device, they have separated inlets, microfluidic network and outlets. Each device was able to generate only three dilution ratios [18]. More recently, Occhetta et al. developed a high-throughput microfluidic screening platform generating six different concentration outflows aimed at 3D cell cultures. Two different variations of linear and logarithmic dilution were created using network channels length as the only variable. The achieved flow rate was 24 μL/h (12 μL/h through every inlet) through the linear dilution device and 45 μL/h through the logarithmic. It is notable that the logarithmic dilution device required a flow ratio between sample and buffer inlets of 1:3.5 [19].

Microfluidic devices are particularly tailored for PoC diagnostics, and for these devices one route to development is to adopt standard assays from the laboratory or “assay track” to create diagnostic kits. There are now several PoC platforms that perform *sample-to-answer* operations [20]. Although they are able to deliver multiple functions in a monolithic manner, meeting the “ASSURED” criteria [21] (**A**ffordable, **S**ensitive, **S**pecific, **U**ser friendly, **R**obust and **R**apid, **E**quipment-free, and **D**eliverable to end-users) proposed by WHO is always a challenge [20,22]. This implies that a successful microfluidic PoC device needs, besides sample and reagents manipulation, to be able to perform the detection and signal processing on chip in the most cost- and space-effective manner possible. An alternative microfluidic-based system relying entirely upon printed circuit board (PCB) manufacturing technology which could comply with most of the ASSURED criteria is the lab-on-PCB (LoPCB) [23,24,25,26,27,28]. The main advantage of the LoPCB technology is that it could provide an analytical platform, where the microfluidic components for sample preparation and reagent manipulation can be integrated with electrochemical sensors and bespoke circuitry, generating a monolithic device [29,30,31,32]. Since there is no need for separate electronics and assay platforms, the required footprint of the whole measuring setup decreases, providing direct, more efficient electrochemical sensing in smaller areas/volume, contrary to, for example, bulky high-sensitivity spectrometric apparatus [33,34]. The combination of both, biochemistry and appropriate electronics on the same platform could reduce noise interference from the various electrical interconnections and as a result may improve the measurement’s signal-to-noise ratio (SNR) [35]. Finally, the high degree of electronics integration, the exceptional accuracy, and the accumulated experience and skills of a mature industrial manufacturing process highlight the great advantage to LoPCB platforms [36,37,38].

Herein, we demonstrate a simple, yet elegant, single layer, stepwise serial diluter that could mitigate most of the aforementioned serial diluter issues and can be implemented on PCB substrate. The flow rate through the diluter allows short assay duration time, i.e., less than 5 min. The unit cell is optimised to generate identical dilution rate when, (a) either the applied pressure at the inlets is the same or (b) the flow rates through both inlets are equal. Therefore, the architecture revolves around pressure and flow rate balanced design. It can also be considered as a modular unit cell that simplifies the fluid input driver to a single source for both, sample and diluent solutions. The dilution ratio remains stable, with tolerance to the input pump instabilities. Hence, a single syringe pump or even a compressed air chamber (e.g., blister packaging) could be employed, reducing the complexity, cost and overall weight and footprint of the final device. The performance of the prototyped devices was characterised using glucose as a proof of concept analyte. Good agreement between simulations and measured results was observed, highlighting the validity of our serial diluter. This novel pressure and flowrate balanced design approach could be an ideal candidate for quantitative PoC tests and may be used as a building block for multistage serial dilution cascade sample preparation components generating diluted samples of known concentrations to derive calibration curves on chip. The designs were initially prototyped in-house on PMMA, in order to validate our hypothesis. Subsequently, they were also implemented on PCB by our industrial partner, using standard commercial techniques.

## 2. Materials and Methods

### 2.1. Pressure and Flow Rate Balanced Unit Cell Design

**Figure 1:**
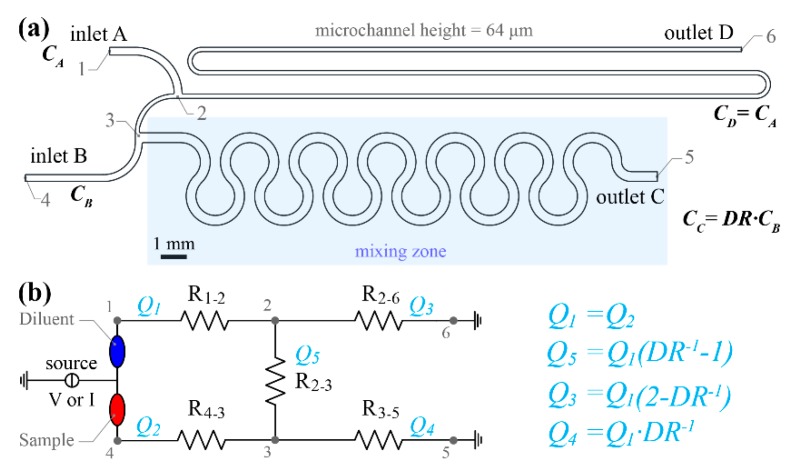
Serial diluter pressure and flow rate balanced unit cell. (**a**) Optimised design to generate dilution ratio *DR* = 2:3. If sample is supplied through inlet B at concentration *C_B_* the concentration of the diluted sample at out C will be *C_C_* = *DR·C_B_* (**b**) Microfluidic network hydraulic resistance analogous electrical circuit. The designed flowrate is 0.4 μL/min. The sample is assumed to enter from inlet B.

Figure 1 illustrates the core design principles of the pressure and flow rate balanced unit cell. The two inlets A and B (see Figure 1a) are designed to be co-linear in pairs with the two outlets (C and D) facilitating the ladder designs to be aligned during lay-out. Inlets A and B can serve both as sample and diluent inlets. This implies that if sample is supplied to inlet B at a concentration *C_B_,* the device will generate a diluted sample at outlet C with concentration *C_C_ = DR·C_B_*. Similarly, if inlet A is selected as sample inlet with concentration *C_A_*, the resulting diluted sample will have a concentration of *C_C_ = (1-DR)·C_A_*. As shown in Figure 1a, the design is divided into four zones. Zones 1 (between points 4 and 3) and 2 (between points 1 and 2) are the device inlets. Zone 3 starts after the branching point 2, where the inlet A stream splits into two sub-streams and ends at point 6 (outlet D). The hydraulic resistance of zone 3 is represented by the notation R_2-6_ in the electrical analogue of the design (see Figure 1b). Zone 4 starts at merging point 3, where sample and diluent streams merge, and ends at point 5 (outlet C). The latter zone is a planar micro-mixing channel consisting of 6 double circular loops. Uniform mixing of the two reagents is crucial for the diluter’s satisfying performance.

Equation (1) can be used for a rough estimation of the required mixing microchannel length considering both, the flow rate and the diffusion coefficient constraints of the given application.

(1) $$Mixing\;Length=u_{avg}\cdot t_{diffusion}=\;u_{avg}\cdot \frac{{\left(cw\right)}^{2}}{2\;D},$$

where *Mixing Length* denotes the estimated required length in metres, *u_avg_* defines the average fluid velocity along the microchannel in m/s, *t_diffusion_* is the required time for a molecule in the sample stream to diffuse across the channel, *cw* is the width of the microfluidic channel in metres, and *D* is the diffusion coefficient of the molecules in the buffer solution in m^2^/s. Equation (1) can be applied to the proposed design, since mixing is mainly driven by molecular diffusion because Reynolds number is low (<100) and consequently the flow field is laminar. Furthermore, any additional convection-driven mixing mechanism induced by the microchannels geometry will only lead to an enhancement of the mixing efficiency. Hence, the result of equation 1 can be always viewed as an *overestimation* of the required mixing length.

Figure 1b presents an electrical analogous circuit of the serial diluter unit cell design of Figure 1a. *Q_1_* is the current (flow rate analogous) through R_1-2_ (inlet A) and *Q_2_* denotes the current through R_4-3_ (inlet B). As an initial step, the two flow rates (or currents) were assumed equal, in order to facilitate the dilution network operation using only one source. The hydraulic resistances R_1-2_, R_2-3_ etc. in this sketch are named after the numbered points illustrated in Figure 1a. *DR* is determined by the flow rate ratio of the two streams joining at the merging point 3. An additional requirement for the design optimisation phase was that the pressure at the two inlets should be equal.

The resulted pressure-balanced unit cell design exhibits several advantages over existing unbalanced ones. More specifically, the novelty of the proposed design lies in the fact that any instabilities or oscillations induced by input flow source fluctuations (due to syringe pump linear speed variation etc.) will not affect the dilution ratio at each stage. This is because only a single flow source is required (e.g., syringe pump) to drive both, sample and diluent (see Figure 1b). As a result, the flow rate oscillations are synchronised (in terms of frequency and phase) and therefore the dilution ratio will be constant. This pressure-balanced design could also operate with a more cost-effective, low-power pneumatic pressure source (either pneumatic pump or compressed air chambers), instead of the traditional syringe pump to drive sample and diluent through the serial diluter. In addition, the two inlets can be *bridged* (as illustrated in Figure 1b) utilising the same pressure source. In this case, any pressure variations at the inlets will again not affect the mixing ratio in every unit cell but will only affect the total flow rate. This implies that the required interfacing ports for the sample and buffer manipulation can be reduced from two to a single one, reducing the final device’s overall size even further. It is worth mentioning that the pressure and flow rate balanced serial dilution network will generate diluted samples only if the liquids are Newtonian fluids of similar viscosity.

**Figure 2:**
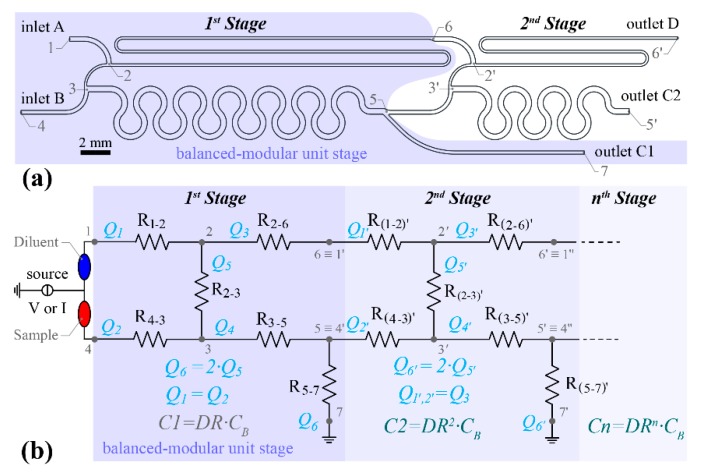
The two-stage stepwise serial diluter of the PCB based device (**a**) design overview highlighting the balanced modular unit stage (**b**) electrical analogous resistor-base network of an n-stage serial diluter design. The dilution ratio per stage is *DR = 2:3*.

Figure 2a demonstrates a two-stage serial diluter design after simulation optimisation, which stems from the combination of two single stage diluters already shown in Figure 1a. Inlet flow rates A and B were again assumed to be equal. The *DR* for both stages has been selected to be 2:3. The flow rate at both inlets of the second stage (point 6 and sampling point 5) is half compared to the flow rate through inlets A and B (see Figure 2b). Due to the pressure balanced design, the pressures at both positions 6 and 5 are equal (see Figure 2b). The hydraulic resistor noted as R_5-7_ defines this pressure. Furthermore, R_5-7_ can be calculated based on the hydraulic resistance of the entire 2nd stage R_tot 1′-6′_, i.e., R_5-7_ = R_tot 1′-6′_·(*2 DR-1)(1-DR)^−1^*. Also, the length of the mixing microchannel between points 3′ and 5′ is shorter (approximately half) than the micro-mixing zone of stage 1. The highlighted 1st stage in Figure 2a,b is the balanced modular unit cell that can be used as building block for cascade designs forming *n-stage* serial diluters. However, this device feature is only valid for liquids (sample and diluent) of similar viscosity. If the diluted sample flowing though branch point 5 is of different viscosity this will affect the pressure drop balance between the microchannels of the 2nd stage and consequently the resulting dilution ratio. It is also worth mentioning that every subsequent stage after the 1st one operates with lower flow rate compared to the previous one (i.e., *Q_1′_,Q_2′_ = Q_3_ = Q_1_ (2-DR^−1^)* see Figure 1 and Figure 2), due to the design architecture and therefore, the mixing efficiency of the micro-mixing zone is expected to be higher than the 1st stage.

### 2.2. Analytical Design, Simulation Model and Optimisation

It is quite common to design complex microfluidic networks based on simplified equations for hydraulic resistance. Such an approach is accurate only when the microchannel cross section is the same throughout the whole device. Microfluidic chips that are based on unique microchannel cross sections usually have very long channels, with high pressure drops and may suffer from flow instabilities, due to material deformation and capacitive effects and extended filling times insufficient for rapid analysis. In this work, we optimised not only all the microchannel lengths but also their widths, based on the desired flow rates. In our case, the use of simplified hydraulic resistance equations for rectangular microchannel is not recommended, due to the deviation from the actual value, which is typically around 20% [39]. A more accurate approximation based on an electric circuit analogy was used as an initial step for the designs, following the methodology described by [39]. The relationship between the volumetric flowrate *Q* and the pressure drop Δ*P* along a perfectly rectangular channel for a steady-state, pressure-driven, fully developed, laminar flow of an incompressible, uniform-viscous Newtonian liquid (aqueous solutions) is well known, assuming that the pressure gradient along the microchannel is uniform, and can be described by the following simplified Hagen–Poiseuille’s law:

(2) $$\Delta P=Q\cdot R_{ch}$$

where *R_ch_* denotes the hydraulic resistance of the microfluidic channel in Pa m^3^ s^−1^. For a rectangular microchannel the hydraulic resistance can be calculated as the summation of a Fourier series [40], where the first six terms of the series are sufficient to calculate *R_ch_*, generating a negligible error of ~10^−6^:

(3) $$R_{ch}=\mu \cdot L\cdot {\left[\frac{A\cdot ch^{2}}{4}\cdot \left(\frac{1}{3}-\frac{ch}{cw}\cdot \frac{64}{\pi^{5}}\sum_{n=1}^{\infty \;\left(practically\;6\right)}\frac{\tanh\left(\frac{cw}{ch}\left(2n-1\right)\frac{\pi }{2}\right)}{{\left(2n-1\right)}^{5}}\right)\right]}^{-1}$$

where *μ* denotes the dynamic viscosity of the liquid in s·Pa (*μ_water_* = 10^−3^ s·Pa), *L* the length of the microfluidic channel, *ch* the channel height, *cw* the channel width and *A* the cross-sectional area of the microchannel (i.e., *A = ch*·*cw*). Equation (2) could be viewed as a hydraulic analogous equation of Ohm’s law. Pressure drop Δ*P* is analogous to the voltage drop Δ*V*, flow rate to the current through the resistor and hydraulic resistance to the electrical resistance. A mass conservation equation in each node can be used, in a complete analogy to Kirchhoff’s current law (KCL). The required number of equations to define the hydraulic resistances of the microfluidic network can be defined using the energy conservation equations, again in complete analogy to Kirchhoff’s voltage law (KVL). On the other hand, equation 3 indicates that the hydraulic resistance is a function of both channel width and length (channel height is defined by the thickness of the dry photoresist (DPR)). The final shape of the microfluidic device was defined after COMSOL Multiphysics^®^ optimisation simulations.

More specifically, using the resulting hydraulic resistances we defined channel widths and lengths based on the technology limitations (minimum feature size i.e., MFS). Moreover, the desired pressure drop along the unit cell to mitigate the interfacing challenges was an additional constraint. The initial topology of the device was constrained by geometrical factors to facilitate the cascade design and minimise the footprint of the device as much as possible. The initial geometry was designed using a parametric 3D CAD software [41] while COMSOL Multiphysics^®^ [42] was used for the 3D modelling, simulation and optimisation of the proposed designs. The numerical model included laminar flow and transport of diluted species models and was solved in a sequential, coupled manner, as shown in previous work [43]. An automated iterative simulation methodology was employed where the parametric 3D model was transferred to the simulation software, that after deriving a converged solution of the flow and pressure field, amended the geometry parameters of the model feeding back the results to the 3D CAD software. This loop was repeated several times until the following two constrains were finally fulfilled:

1. the flow rate boundary conditions as presented in Figure 1b (or Figure 2b) at the inlets and outlets of the network,
2. the absolute average pressure difference between the inlet surfaces A and B should be less than 0.01 Pa (see Figure 1b and Figure 2b).

The diffusion coefficient of the sample in the buffer solution was assumed to be 6.67 10^−10^ m^2^/s (Glucose in aqueous solutions) [44]. For the PCB-based serial diluter, both optimised designs (the balanced unit cell shown in Figure 1 and the two-stage serial diluter in Figure 2) were transferred to Altium Designer [45], a commercially available software used for PCB design and layout. The produced standard Gerber files were then submitted to an industrial PCB manufacturer, Newbury Electronics Ltd. for fabrication.

#### 2.2.1. Laminar Flow Model

In this work a laminar incompressible steady state flow model has been assumed. The medium flowing through the channel via both inlets was selected to be water at 20 °C. Considering the kinematic viscosity of the fluid, design flow rates have Reynolds number within the laminar range (Re <<100). The model takes into consideration mass and momentum conservation equations for steady state incompressible flow, where gravitational effects are neglected. The equations can be seen below:

(4) ∇⋅u=0

(5) $$\left(u\cdot \nabla \right)u=-\frac{\nabla p}{\rho }+\nu \nabla^{2}u$$

with ***u*** denoting the velocity vector, *p* the pressure, *ρ* the density of the medium and *ν* the kinematic viscosity [46]. A parabolic velocity profile of fully developed laminar flow was formed over the two inlets. No-slip velocity on the channel walls was used as a boundary condition. Pressure at every device outlet was defined equal to 0 Pa (system boundary conditions). The dilution networks were numerically investigated for the following flow rates in case of the PCB based prototypes: 0.2 μL/min, 0.4 μL/min, 0.6 μL/min and 0.8 μL/min. The PMMA prototypes were investigated numerically at 10 μL/min.

#### 2.2.2. Diffusion Model

The concentration-based advection-diffusion model of COMSOL Multiphysics^®^ (COMSOL, Inc., Burlington, MA 01803, USA ) was employed for mixing efficiency studies. The mass balance equation describing the steady-state problem is described by the following relation:

(6) u⋅∇C=∇⋅(D∇C)

where *C* is the concentration of the diluted species in mol/L. The concentration of diluted species over inlets A and B, in both designs, was set equal to 0.00 and 1.00 mol/L, respectively, i.e., *C_A_* = 0.00 mol/L and *C_B_* = 1.00 mol/L. At this point, the interested reader should note that any correlation between the fluid kinematic viscosity or density and the glucose concentration has been neglected.

#### 2.2.3. Mixing Efficiency

Throughout the literature several micromixer performance quantification methods are proposed [47,48,49]. Since adequate mixing is defined as the homogeneity of the mixed components, the distribution of the concentration over the computational nodes of a flow cross-section can be used to evaluate the degree of mixing. Nguyen proposed the Mixing Efficiency (ME) [49] as a mixing quantification parameter with the following mathematical expression:

(7) $$ME=1-\sqrt{\frac{1}{N}\overset{N}{\underset{i=1}{\sum }}{\left(\frac{C_{i}-\bar{C}\;}{\bar{C}}\right)}^{2}}$$

where $\bar{C}$ is the concentration of fully mixed medium (in case of *DR = 2:3*, $\bar{C_{1}}$ = 0.67 mol/L and $\bar{C_{2}}$ = 0.44 mol/L), $C_{i}$ is the concentration at a given position (spatial discretisation point) and *N* is the total number of discretisation points over the outflow surface. In other words, equation 7 is a normalised standard deviation of the diluted species concentration over the computational nodes, with values ranging from 0.00 to 1.00 (0.00 defining completely unmixed species and 1.00 an ideally uniform mixed flow cross-section) [49]. In this work, the ME has been calculated on the computational nodes of the outflow surface.

### 2.3. Device Fabrication

#### 2.3.1. PCB Based Devices

The commercially fabricated microfluidic PCB devices comprises a standard FR-4 sheet (Flame Retardant grade 4, Newbury Electronics Ltd., Newbury, Berkshire, RG14 2AD, UK ) and a 64 μm thick DPR, patterned using standard lithography employed in commercial PCB manufacturing technology. A laser direct imaging system (Limata Gmbh, UV-P300 LDI, Ismaning, Germany) was used to pattern the DPR. This fabrication method offers maximum sidewall roughness ± 10% of the DPR resolution (typically DPR thickness), i.e., ± 6.4 μm. The final device comprises the PCB based microfluidic layer on the bottom (FR-4 and DPR) and two additional layers of polymethyl methacrylate (PMMA) to create the microfluidics interface (in house fabrication). A detailed fabrication process and an exploded view of the fabricated device is in the supplementary materials (Figure S1). The devices were sealed using polyethylene terephthalate (PET) sealing film (50 μm thickness, PET, VWR^®^ Polyester Sealing Films for ELISA). After opening sample inlet and outlet holes on the PET film using a CO_2_ laser cutter (Mini 24 Legend Laser System, Epilog Laser, Golden, CO 80403-1826, USA), both the PCB microfluidic network and the PET film were placed in a pouch laminator (60 C, lamination speed around 4 mm/s). A double-sided adhesive 127 μm thick film (3M™ High Performance Acrylic Adhesive 200MP) was used to bond the additional PMMA tubing interfacing layer (room temperature, lamination speed around 4 mm/s). Inlet and outlet ports were opened using again the laser cutter. 40 kPa pressure was applied for 1h to bond the two components together at room temperature.

#### 2.3.2. PMMA Based Devices

The PMMA based devices were designed and prototyped following the same principle as described previously, but with much bigger footprint generating *DR = 5:6*. The PMMA rapid prototyping involves the CO_2_ laser micromachining of a 175 μm PMMA sheet (Goodfellow PMMA, Acrylic sheet), stacked between two adhesive layers each one 50 μm thick (3M™ High Performance Acrylic Adhesive 200MP). The stack was formed employing a pouch laminator (60 °C, lamination speed around 4 mm/s). The microfluidic channels were cut through the stack using the CO_2_ laser cutter. Bottom (1 mm thick) and top (3 mm thick) PMMA layers (clear extruded PMMA sheets, Perspex^®^, Darwen, Lancashire, UK), were machined to allow for device support and tubing interfacing respectively. The stack was bonded together after alignment with a pressure of 40 kPa for 1h at room temperature. Fabrication steps and an exploded view of the fabricated device is in supplementary materials (Figure S2).

## 3. Results and Discussion

### 3.1. Computational Results

#### 3.1.1. First-Cut Approximation

The first stage of the electrical analogous circuit illustrated in Figure 2b was used to define the required hydraulic resistances of the pressure and flow rate balanced modular unit cell. The network comprises five nodes (see Figure 2b positions 1–7) and 6 hydraulic resistances, R_1-2_ R_2-3_ R_2-6_ R_4-3_ R_3-5_ and R_5-7_. The desired *DR* of this unit cell has been set equal to 2:3, while the nominal flowrate of the device was defined as 0.4 μL/min after some iteration to facilitate sample formation at the outlets within a reasonable operating time (less than 3 min). Applying the mass conservation equation in nodes 2 and 3, we derive the following equations:

(8) $$Q_{3}=Q_{1}-Q_{5}$$

(9) $$Q_{4}=Q_{1}+Q_{5}$$

The flow rate balanced design satisfies the following 2 equations

(10) $$Q_{1}=Q_{2}$$

(11) $$Q_{6}=Q_{4}-Q_{3}\overset{\left(8\right),\left(9\right)}{\Leftrightarrow }Q_{6}=2\cdot Q_{5}$$

The required dilution ratio (*DR = 2:3*) complies with the following equation

(12) $$Q_{5}=Q_{1}\cdot \left(\frac{1}{DR}-1\right)$$

From Equations (8)–(12) with the desired device specifications (*DR* and nominal flow rate), all flowrates in the modular unit cell can be computed. The minimum feature size of PCB technology is 150 μm (MFS = 150 μm) due to the DPR technical specifications. Both inlet microchannels were initially assumed to have the same channel width equal to double the MFS, i.e., *cw*_1-2_ = *cw*_4-3_ = 2 MFS = 300 μm. The channel width of the microchannel between positions 2 and 3 was selected to be slightly wider than the MFS, *cw*_2-3_ =160 μm while its length was selected arbitrary as L_2-3_ = 2.5 mm. The mixing channel between position 3–5 was selected to be wider than the inlet channels since the flowrate though it is 50% higher than the nominal flowrate (*DR = 2:3*), *cw*_3-5_ =380 μm. Equation (1) can be used as a rough estimate of the mixing zone channel length. The employed mixing length was increased by 30% as factor of safety to assure sufficient mixing efficiency, resulting in 58 mm. The bypass diluent microchannel width between positions 2 and 6 has been set as *cw*_3-5_ = 170 μm, while the outflow channel was selected initially to have a width of 300 μm as the device inlets. However, after the optimisation study the final width was defined to be 220 μm, so that the resulting channel length facilitates the sampling position to be at certain distance from the dilution network (facilitating the device interfacing as well).

The pressure and flowrate balanced design approach together with the requirement for low pressure drop is defined by the following two equations.

(13) $$P_{1}=P_{4}=110\;Pa$$

(14) $$P_{6}=P_{5}=10\;Pa$$

Using Equation (3) the hydraulic resistances of branches *R_2-3_* and *R_3-5_* can be computed. The four remaining hydraulic resistances (*R_1-2_*, *R_4-3_*, *R_2-5_* and *R_5-7_* can be determined after solving the following linear equations system. These equations are complying with the conservation of energy law along closed paths through the microfluidic networks (in accordance with Kirchhoff’s voltage law), summarised below:

(15) $$Q_{1}\cdot R_{1-2}=Q_{2}\cdot R_{4-3}-Q_{5}\cdot R_{2-3}$$

(16) $$Q_{3}\cdot R_{2-6}=Q_{5}\cdot R_{2-3}+Q_{4}\cdot R_{3-5}$$

(17) $$P_{1}-P_{7}=Q_{2}\cdot R_{4-3}+Q_{4}\cdot R_{3-5}+Q_{6}\cdot R_{5-7}$$

(18) $$P_{1}-P_{6}=Q_{1}\cdot R_{1-2}+Q_{3}\cdot R_{2-6}$$

This method can be applied to design pressure and flowrate balanced modular unit cells of various dilution ratios by modifying the *DR* parameter. The hydraulic network illustrated in Figure 2b can be designed in the same way if the hydraulic resistance R_5-7_ is zero. After obtaining the values of the hydraulic resistances, using Equation (3) the microchannel lengths can be calculated. A Matlab^®^ custom-made script has been written for the solution of the system shown in Equations (15)–(18) and the computation of the various hydraulic resistances and microchannel lengths (Equations (1)–(3)). Matlab^®^ numerical results from the first-cut approximation method are shown later together with COMSOL^®^ optimisation results.

#### 3.1.2. Simulation and Optimisation

##### Single-Stage Diluter

**Figure 3:**
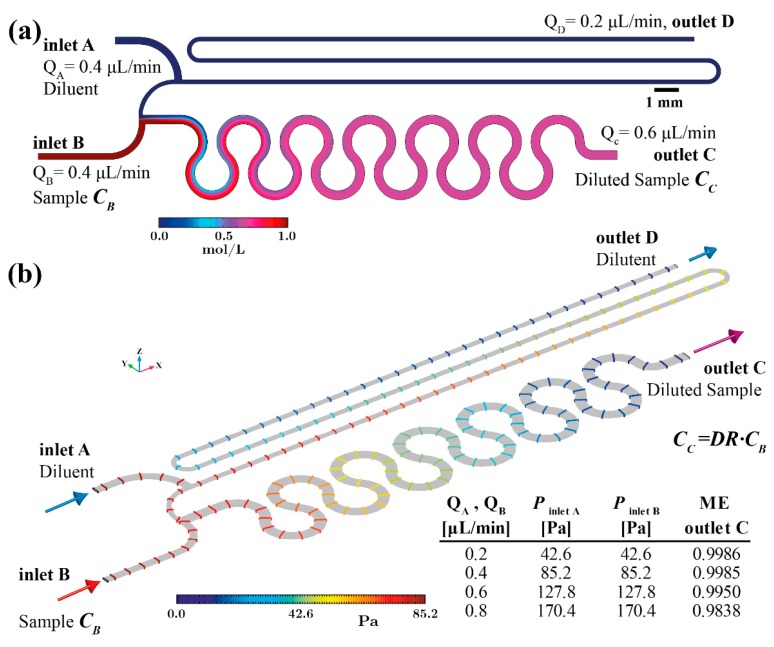
Pressure and flow rate balanced unit cell simulation results (**a**) concentration field overview on the device symmetry *x*-*y* plane (*z* = 32 μm) (**b**) iso-pressure lines overview in case of inlet flow rate 0.4 μL/min and *DR* 2:3.

Figure 3 summarises the computational results of the PCB based device performance when the flow rate through both inlets (A and B) equals 0.4 μL/min. This is the nominal flow rate of the PCB device that offers sufficient time for the diluted sample to be formed at the device outlet (less than 3 min, since the optimised unit cell volume is 2.32 μL), i.e., steady state.

The concentration field in Figure 3a verifies that the six double-loop micromixing channel has adequate length to achieve uniform mixing at the device outlet. In Figure 3b the pressure field along the device with flow rate 0.4 μL/min is shown. The coloured iso-pressure lines illustrate the positions experiencing equal pressure values. In addition, the table presented in Figure 3b summarises the pressure at the inlet surfaces for various flow rates. Evidently, the pressure balanced optimised design results in equal pressure formation on both inlets for every simulated flow rate. More specifically, the nominal inlet flow rate (0.4 μL/min) through both inlets generates 85.2 Pa. This order of pressure magnitude can be easily provided by a burst blister packaging. Furthermore, the pressure created on both inlet surfaces increases in proportion to the flow rate while remaining equal. Consequently, this design offers dilution rate performance that is tolerant of flow source fluctuations. However, as indicated in the table of Figure 3b, for flow rates higher than 0.6 μL/min the ME drops (ME = 0.98 for *Q_A_,_B_* = 0.8 μL/min).

##### The Two-Stage Serial Diluter

In Table 1, columns 2 and 3, we summarise the results of the “first-cut” approximation numerical simulation based on the Matlab^®^ code for the PCB-based device. These microchannel lengths are not the final, fabricated ones. These values were used to define an initial design of the network that was then used in an automated optimisation study using Solidworks^®^ (Dassault Systèmes SolidWorks Corporation, Waltham, MA 02451, USA) and COMSOL Multiphysics^®^ (COMSOL, Inc., Burlington, MA 01803, USA) in a coupled manner. The final geometrical parameters of the modular unit cell (Table 1, columns 4 and 5) were used for the fabricated and characterised devices that will be presented later. To compare the analytical calculations based on equations (1)–(3) and (8)–(18) with the *first-cut* approximation numerical simulation, we used the optimised microchannel widths (Table 1, column 4) and the resulting pressure values of the simulation results as input to solve the system of equations. Table 1, column 6 presents the obtained microchannel lengths provided by the Matlab^®^ script. Comparing the microchannel lengths in column 5 and 6 of Table 1, it can be seen that the *first-cut* approximation method results from Matlab^®^ (MathWorks^®^, Natick, MA 01760-2098, USA) are in good agreement with COMSOL^®^ (COMSOL, Inc., Burlington, MA 01803, USA) final optimised design results.

**Figure 4:**
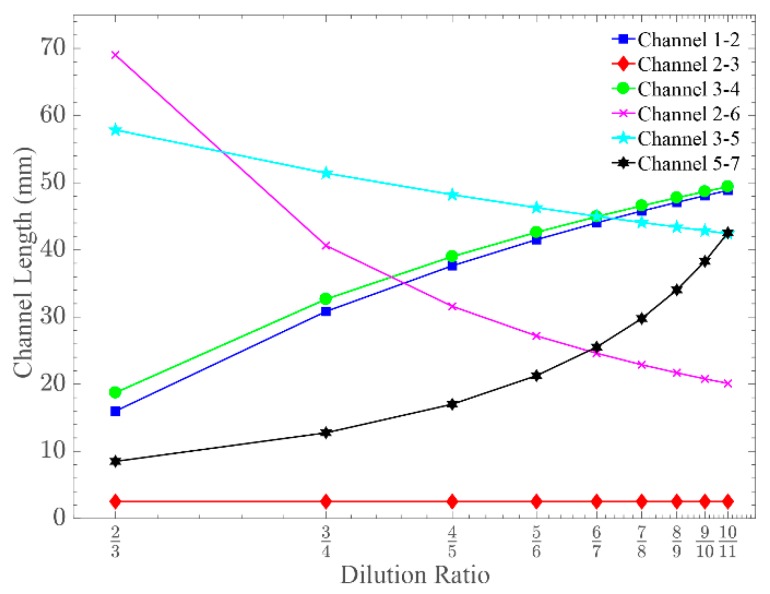
The design rules of the pressure and flow rate balanced modular unit cell showing the required microchannel lengths for various dilution ratios. Microchannel widths are kept the same as the optimised modular unit cell-generating dilution ratio of 2:3.

Finally, Figure 4 summarises the design rules of the modular pressure and flow rate balanced unit cell of Figure 2b for various dilution ratios, ranging from 2:3 to 10:11, based on the custom Matlab^®^ script. All the solutions were derived with constant channels widths, equal to the computationally optimised ones (see Table 1 column 4).

**Figure 5:**
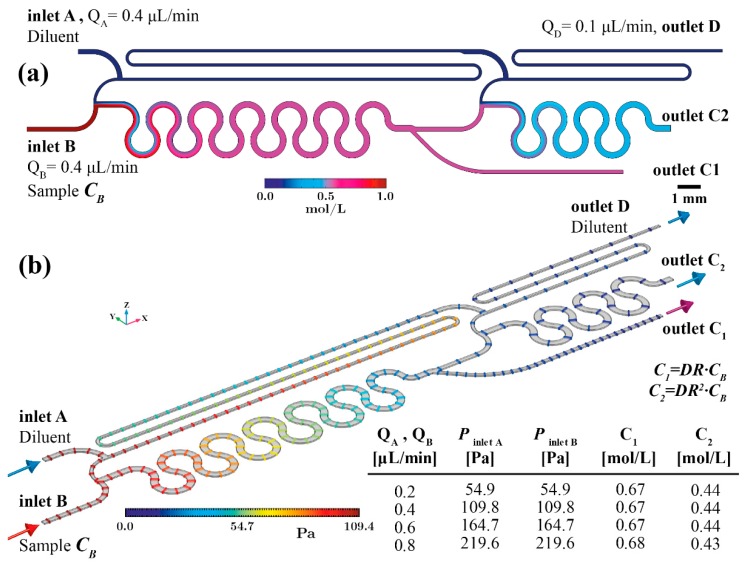
Pressure and flow rate balanced PCB two-stage serial diluter simulation results. (**a**) concentration field overview on the device symmetry x-y plane (z = 32 μm) (**b**) iso-pressure surfaces. The table summarises the average pressure at the inlets and the average concertation values at the outlets for various flow rates.

In Figure 5a computational results for the concentration field on the z-axis midplane of the device are plotted with the nominal flow rate (0.4 μL/min) applied through both inlets (A and B). The volume of the PCB-based two-stage diluter is 3.87 μL and consequently less than 5 min is required to generate the two diluted sample concentrations (i.e., *C_1_* = 2:3 *C_B_* and *C_2_* = 2:3 *C_1_*). In order to minimise the device footprint (105 × 8 mm^2^) the 2nd stage mixing zone (between point 3′ and 5′) comprises a three double-loop microchannel. It can be seen that the achieved mixing efficiency of every stage allows for dilution rate generation according to the designs (see table in Figure 5b).

Finally, Figure 5b shows the pressure drop along the PCB two-stage diluter. Additionally, the inclusive table of Figure 5b summarises the computational results for the pressure on the inlet surfaces for the various flow rates. The iso-pressure lines in Figure 5b along the two-stage diluter visualise the positions where the pressure level is the same. Evidently, the optimised two-stage design results in equal pressure on both inlets for every flow rate. It was computed that pressure equal to 109.8 Pa is required to achieve the nominal inlet flow rate (0.4 μL/min) through both inlets.

Due to the design optimisation objectives, if the applied pressure on both inlet surface increases, the flow rate increases proportionally while the generated diluted samples concentrations (*C_1_* and *C_2_*) remain unaffected. However, for flow rates higher than 0.6 μL/min the ME drops, resulting in a non-uniform diluted sample concentration.

Consequently, the dilution ratio is affected for both outlets. In detail, for the case of 0.8 μL/min inlets flow rate, the resulting *C_1_* outlet sample concentration is *C_1_* = 0.68 mol/L. Thus, the generated dilution ratio is 0.68 instead of 0.67, which is the design dilution ratio of both stages (*C_1_* = 2:3 *C_B_* or 0.67 *C_B_*). This minor deviation is attributed to the fact that the sampling microchannel (see Figure 2a, between pos. 5–7) at position 5 is placed on the same side as the sample inlet (inlet B). As a result, the non-uniformly generated diluted sample gives a higher concentration on the inlet B side. The 2nd stage is provided with lower concentration inlet sample and the diluted sample through outlet *C_2_* presents lower concentration (i.e., *C_2_* = 0.43 mol/L instead of 0.44 mol/L in case of nominal inlet flow rate).

### 3.2. Experimental Performance and Validation

#### 3.2.1. PMMA Prototype: Dilution Ratio Stability and Performance Validation

**Figure 6:**
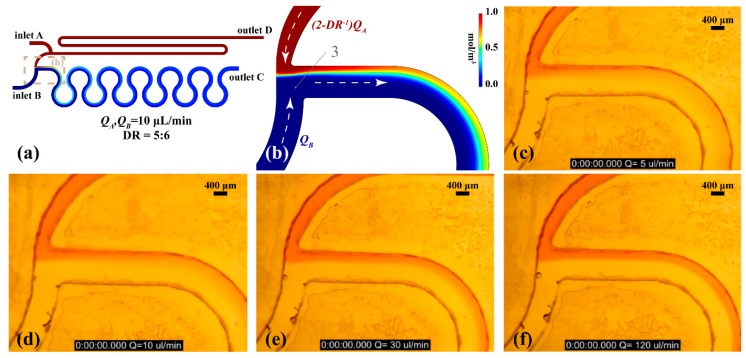
Serial diluter unit cell prototyped on PMMA. (**a**) design top view. Simulation results of the concentration field (**b**) detail of the concentration field at the merging point 3. Sample inlet and buffer inlet flow rates equal to (**c**) 5 μL/min (**d**) 10 μL/min (**e**) 30 μL/min (**f**) 120 μL/min.

The effectiveness of the pressure and flowrate balanced design on dilution ratio was investigated for the PMMA prototype. This device is transparent allowing easy observation with a microscope, enabling validation of the dilution ratio stability. Figure 6a shows the concentration field of the optimised diluter unit cell for the PMMA device. *DR* is 5:6 while inlet flowrates *Q_A_* and *Q_B_* were 10 μL/min. Figure 6b presents a detail of the concentration field around the merging point 3. The same field of view of the prototype PMMA device is shown in Figure 6c to f during dilution ratio stability characterisation experiments under a wide range of flowrates (from 5 μL/min to 120 μL/min).

A solution of DI water and food colouring was used as inlet A fluid while DI water was driven through inlet B. Both flow rates were equal and were achieved by using a syringe pump loaded with two 10 mL plastic syringes. As illustrated in Figure 6d, the simulation result is in agreement with the former (Figure 6b). Comparing the two figures, it can be observed that the ratio between the red and the blue stream in Figure 6b is approximately equal to the ratio between the two streams in the experimental results (Figure 6d). Figure 6c–f illustrate the stable dilution ratio of the unit cell for a wide range of flow rates confirming the theoretical model since the ratio between the two stream widths is exactly the same in every image.

**Figure 7:**
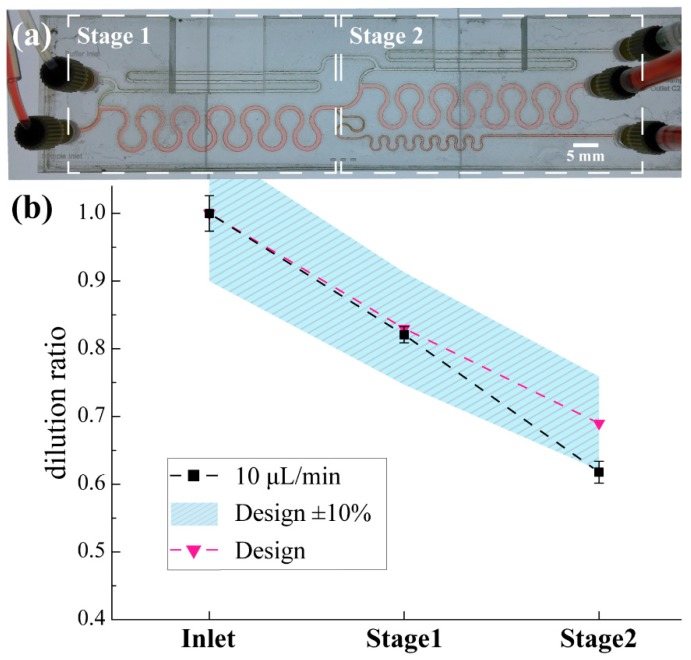
Dilution ratio validation of the two-stage PMMA prototype using commercial Accu-Check^®^ glucose meter. MFS 430μm (**a**) The prototyped two-stage PMMA diluter during performance validation experiment. Channel height is 275 μm (**b**) The solid marks represent the dilution performance under 10 μL/min flow rate.

The performance of the PMMA two-stage serial diluter designs was validated using a commercial glucose meter (Accu-Check^®^ Aviva, F. Hoffmann-La Roche AG, Basel, Switzerland). More specifically, 0.1 M Phosphate-buffered saline (PBS) (inlet A) and 15 mM glucose (Sigma-Aldrich) in PBS (inlet B) were used as diluent and sample solutions respectively. The PMMA prototype was characterised for the design flowrate (10 μL/min). Figure 7a shows the PMMA prototype where the sample glucose solution (inlet B see Figure 2a) includes red food colouring to enable the observation of the device operation.

The design *DR* for every stage is 5:6. Figure 7b summarises the statistically analysed measurements. The average achieved dilution ratio is presented for different sampling positions. The error bars express the standard deviation limits. A satisfactory agreement between the design and experimental data is demonstrated within the expected measurement error (±10%) due to the inherent accuracy of the commercial glucose sensor (Aviva, Accu-Check^®^, F. Hoffmann-La Roche AG, Basel, Switzerland.). Furthermore, it is known that PMMA microfluidic devices fabricated by CO_2_ laser machining in open air conditions exhibit dimensional variation [50]. This variation was non-uniform throughout the device mainly due to the variable optical path of the laser beam and the limitations of the machine in terms of beam motion and power modulation.

#### 3.2.2. PCB-Based Microfluidic Prototype: Dilution Ratio Performance Validation

**Figure 8:**
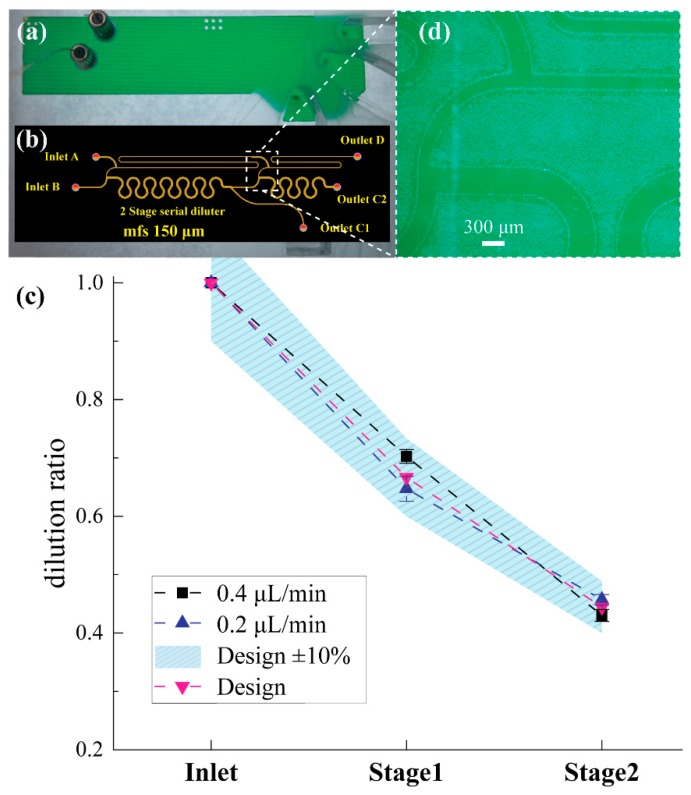
Dilution ratio validation of the two-stage PCB prototype using commercial Aviva Accu-Check^®^ glucose meter. (**a**) The prototyped two-stage PCB diluter during performance validation experiment (**b**) design used for the prototype fabrication. (**c**) The solid marks represent the dilution performance under 0.4 and 0.2 μL/min flow rate (**d**) detail of the fabricated prototype after sealing fabrication step around the merging point of stage 2 (the device is empty).

The performance of the optimised PCB based microfluidic serial dilution network was characterised under two different flow rates, i.e., 0.2 μL/min and 0.4 μL/min. Each of the generated diluted samples (*C_1_* and *C_2_*) was measured three times. Figure 8a shows the top view of the PCB prototyped device during performance characterisation experiments. Figure 8b illustrates the superposition of the various PCB layers (optimised microfluidic network and inlet outlet VIAs) as designed in Altium Designer^®^ (Altium LLC, La Jolla, CA 92037, USA). Figure 8d presents a detail around the merging point of the second stage (see position 3′ in Figure 2a) of the sealed microfluidic PCB device. The statistically analysed dilution ratio measurements are presented in Figure 8c. Rectangular and triangle marks pointing upwards report the measured dilution ratios using the glucose meter, while the design relevant triangular marks pointing downwards are provided for comparison. The highlighted area marking is provided as the acceptable measurement error due to the glucose meter accuracy (± 10%). The error bars indicate the standard deviations for each measured sample. In the case of the 0.2 μL/min inlet flow rate, the 1st and 2nd stage dilution ratio was found to be equal to 0.65 ± 0.02 and 0.46 ± 0.01, in good agreement with the design values, taking also into consideration the glucose meter accuracy. Likewise, for the nominal flow rate (0.4 μL/min) the dilution ratio performance of both stages was found equal to 0.70 ± 0.01 and 0.43 ± 0.01, respectively. It is evident that the device can generate diluted samples of constant concentrations even if the inlet flow rates are fluctuating between the values 0.2 μL/min and 0.4 μL/min. The performance of the PCB based device was not affected by the dimensional variation of the microfluidic network, mainly because the PCB manufacturing technology offers μm-scale dimensional tolerance (e.g., ±6 μm) throughout the entire device. The network comprises microfluidic channels of mm-scale length and 150 μm width. Therefore, non-uniformities in μm-scale dimensional variations do not affect the overall performance.

The sampling point 5 (see Figure 2a) is on the same side as inlet B (point 4). If the flowrate through the micromixing zone (between point 3 and 5) is higher than assumed in the design calculations (*Q_4_ = Q_1_/DR =* 0.6 μL/min) the microchannel length does not allow for uniform mixing of sample and diluent. In parallel, inlet B is used as sample inlet and consequently, the sample stream flowing along the sidewall of the micromixing zone (that is on the same side with the sampling channel, branch 5 to 7 in Figure 2) is not fully diluted. Therefore, the liquid flowing through the outlet C1 is expected to have higher concentration than designed. The table inset in Figure 5b shows that for the case of *Q_A_* = *Q_B_* = 0.8 μL/min, the resulting concentration in outlet C_1_ is 0.68 mol/L instead of the 0.67 mol/L. Additional experiments with higher flow rates (i.e., 0.8 μL/min) resulted in dilution performance outside the design values, as predicted by the simulation results shown in Figure 5b.

A direct comparison between the experimental results obtained using the PMMA and PCB prototypes shown in Figure 7 and Figure 8, respectively, reveals that the PCB-based serial diluter demonstrates higher reproducibility compared to the PMMA one, with its diluted output samples been in good agreement with the ideal, theoretical values expected from the designs. The reproducibility and accuracy demonstrated by the PCB-based diluter is mainly due to the dimensional accuracy and uniform geometrical variations guaranteed by the industrial PCB manufacturer. The PMMA prototypes have been developed using a CO_2_ laser in open air micromachining conditions with fabrication variations significantly higher compared to the mature PCB ones. This superior performance of the PCB diluter enhances our initial argument regarding the advantages of the LoPCB paradigm, which relies entirely on this established manufacturing method that could ensure minimal fabrication variations in the serial dilution and the biosensing stages of a PCB-based PoC testing platform.

## 4. Conclusions

In this work a modular serial dilution unit cell has been conceived, designed, optimised and fabricated, offering both pressure and flow rate balance. The designs comprising the modular unit cell were optimised using Solidworks^®^ (Dassault Systèmes SolidWorks Corporation, Waltham, MA 02451, USA) and COMSOL Multiphysics^®^ (COMSOL, Inc., Burlington, MA 01803, USA) with a laminar flow and diffusion-advection model. The PMMA based single stage (unit cell) design was prototyped in house and was used for dilution ratio stability validation experiments for a wide range of flow rates. The two-stage step-wise serial dilution networks was designed and optimised for two substrates; PMMA and PCB. The first was designed to generate diluted samples with dilution ratio equal to 5:6 per stage and the later 2:3. The optimised PMMA designs were prototyped *in-house* while the PCB microfluidic networks were fabricated by a commercial PCB manufacturer. Device performance was validated experimentally using glucose. Simulation results were in good agreement with the measured results.

If required, the modular serial dilution unit cell can be repeated in a cascade manner to form multistage serial dilution networks of compact footprint (around 75 × 8 mm^2^ per stage), while the pressure and flow rate balanced capability minimises the number of flow sources. Both diluent and sample can be driven through the device using a single flow source. However, the trade-off for the modular pressure and flowrate balanced design is that the flowrate of diluted sample and diluent entering each subsequent stage is much lower than the previous, i.e., *Q_1′_ = Q_1_·(2−DR^−1^)* resulting in extended time for the diluted samples to appear at the outlets. The low pressure required at both inlets (109.8 Pa or 11.2 mm H_2_O) guarantees the above. In addition, the dilution ratios tolerate flow source instabilities, since the dilution ratio remains constant as long as the nominal flow rate or inlet pressure is not exceeded.

The proposed design is an ideal candidate for affordable PoC platforms relying on quantitative assays, where for example, serial dilutions of a known sample concentration are required so that a standard curve can be used as a reference. The modular unit cell can be cascaded for more complicated multistage serial dilution network applications and is compatible with PCB-based biosensors and electronic components, allowing for the monolithic integration on a PCB substrate, when quantitative PoC medical diagnostic tests are required.

## Figures and Tables

**Table 1:** Microfluidic network geometrical parameter results for the modular pressure and flow rated balanced unit cell.

Channel	Matlab^®^ *first-cut* Approximation *P_1_-P_6_ = 100 Pa, P_1_ = 110 Pa*		COMSOL^®^ Optimisation Results *P_1_-P_6_ = 85.4 Pa, P_1_ = 109.8 Pa*		Matlab^®^ Validation Results *P_1_-P_6_ = 85.4 Pa, P_1_ = 109.8 Pa*
	Width *cw* [μm]	Length *L* [mm]	Width *cw* [μm]	Length *L* Optimization Study [mm]	Length *L* Validation Calculations[mm]
1-2	*2 MFS = 300*	*16.0*	*2 MFS = 300*	*3.7*	*3.4*
4-3	*2 MFS = 300*	*18.7*	*263*	*5.4*	*5.3*
2-3	*160*	*2.5*	*160*	*2.5*	*2.5*
2-6	*170*	*69.0*	*170*	*67.5*	*69.0*
3-5	*380*	*57.9*	*380*	*57.9*	*57.9*
5-7	*2 MFS = 300*	*8.5*	*220*	*14.4*	*14.5*

## Data Access Statement

The data from this paper can be obtained from University of Southampton Institutional Repository (https://doi.org/10.5258/SOTON/D0810).

## Supplementary Materials

The following are available online at https://www.mdpi.com/1424-8220/19/4/911/s1, Figure S1: title, Table S1: title, Video S1: title.
