# Supplementary material for: Modular Pressure and Flow Rate-Balanced Microfluidic Serial Dilution Networks for Miniaturised Point-of-Care Diagnostic Platforms
Source: Sensors (Basel). 2019 Feb 21;19(4):911. doi: 10.3390/s19040911 (PMC6412972; doi:10.3390/s19040911)
Supplement: Supplementary file 1 [file sensors-19-00911-s001.pdf]

sensors

MDPI

Article ESI

Modular Pressure And Flow Rate Balanced Microfluidic Serial Dilution Networks for Miniaturised Point-Of-Care Diagnostic Platforms

Nikolaos Vasilakis [1,*], Konstantinos I. Papadimitriou [1], Hywel Morgan [1,2] and Themistoklis Prodromakis [3]

[1] Nanoelectronics & Nanotechnology Research Group, Electronics and Computer Science, University of Southampton, Southampton, SO17 1BJ, UK
[2] Institute for Life Sciences, University of Southampton, Southampton, SO17 1BJ, UK
[3] Zepler Institute for Photonics and Nanoelectronics, University of Southampton, Southampton, SO17 1BJ, UK
* Correspondence: n.vasilakis@soton.ac.uk;

Received: date; Accepted: date; Published: date

PCB-based device fabrication process:

In Figure 1S the various layers that comprise the PCB-based device are illustrated. The fabrication procedure is as follows:

1. The PCB microfluidic network (rigid FR-4 substrate and lithographically patterned DPR) was fabricated by our industrial partner (Newbury Electronics Ltd) utilising standard PCB manufacturing techniques.
2. A PET film (50 μm thickness, PET, VWR ® Polyester Sealing Films for ELISA) was machined (opening inlet and outlet ports) using the CO_2 laser cutter (Epilog Mini 24 Legend Laser System, USA) and subsequently both PCB microfluidic network and PET film were placed in a pouch laminator (60 ºC, lamination speed around 4 mm/s).
3. The two interfacing PMMA layers (3 mm thick PMMA 1, and 5 mm thick PMMA 2 as shown in SF1) were prepared in two steps:
    a. A double-sided (DS) adhesive 127 μm thick film (3M™ High Performance Acrylic Adhesive 200MP) was laminated on the PMMA surface employing the pouch laminator (room temperature, lamination speed around 4 mm/s).
    b. The stack (PMMA and DS) was milled using the CO_2 laser cutter opening inlet and outlet ports.
4. Finally, the two interfacing layers (PMMA 1 – DS and PMMA 2 – DS) where aligned and stacked on top of the sealed PCB microfluidic device. A 40 kPa constant pressure was applied for 1h to bond the components together at room temperature.

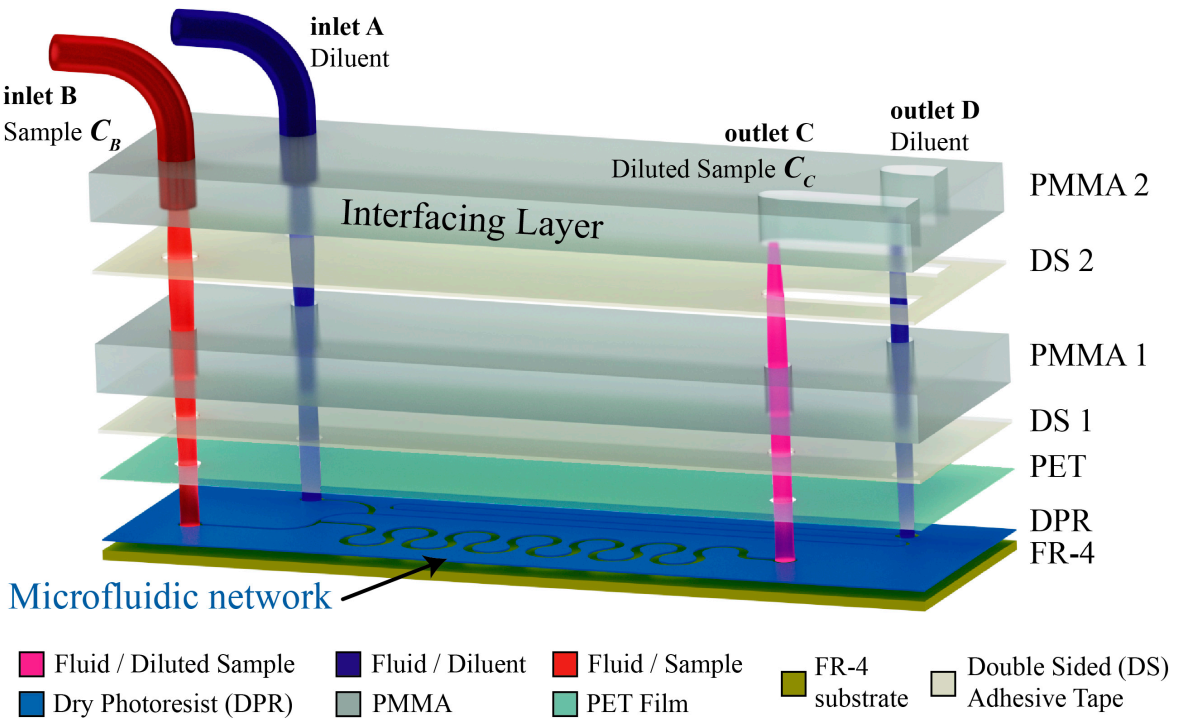

Figure 1S: Exploded view presenting the various PMMA, PET and double sided adhesive tape (DS) layers used to interface the PCB fabricated prototype with the required tubing for the dilution performance characterisation experiments. Both outlets C and D supply the two wells with diluted sample and bypassing diluent respectively.

PMMA device fabrication process:

In Figure 2S the various layers that comprise the PMMA-based device are demonstrated. More specifically, the PMMA microfluidic network comprising 3 PMMA sheets and two double sided adhesive (DS) layers (see Figure SF2a and b, layers PMMA 1 – DS 1 – thin PMMA – DS 2– PMMA 2) are shown in detail in exploded view and the device's fabrication steps are summarised below:

1. Using a pouch laminator, the stack illustrated in Figure SF2b was formed. Initially, the first DS 1 layer (50μm thick 3M™ High Performance Acrylic Adhesive 200MP) was laminated on one side of the thin PMMA sheet (175 μm thick Goodfellow PMMA Acrylic sheet). For this step a pouch laminator was employed (60°C, lamination speed around 4 mm/s). Subsequently, the second DS layer (DS 2) was laminated on the other side of the thin PMMA sheet using again the pouch laminator (same lamination speed and temperature conditions).
2. The DS 1-thin PMMA-DS 2 stack was micromachined using the CO_2 laser cutter. The stack was placed on top of a PMMA sacrificial layer to facilitate the laser ablation process. The microfluidic network was cut through the 3-layer stack (Figure SF2b).
3. Two PMMA layers (see Figure SF2, 1 mm thick PMMA 1 and 3 mm thick PMMA 2) were machined using the CO_2 laser cutter.
4. The three fabricated components (PMMA 1, DS 1-thin PMMA-DS 2 and PMMA 2) were aligned and bonded together by applying 40 kPa constant pressure for 1h at room temperature.

The top PMMA interfacing layer (see Figure SF2, 3 mm thick PMMA 3 and DS 3) was fabricated in two steps:

a. A DS adhesive 127 μm thick film (3M™ High Performance Acrylic Adhesive 200MP) was laminated on the PMMA surface employing the pouch laminator (room temperature, lamination speed ~ 4 mm/s).

b. The stack (see Figure SFa, PMMA 3 and DS 3) was milled using the CO_2 laser cutter opening inlet and outlet ports.

The final step of the PMMA-based prototype required the top interfacing PMMA layer (Figure SF2a, PMMA 3 and DS 3, fabrication steps 5 and 6) and the microfluidic device (fabrication steps 1 to 4) to be aligned and stacked. A 40 kPa constant pressure was applied for 1h to bond the components together at room temperature.

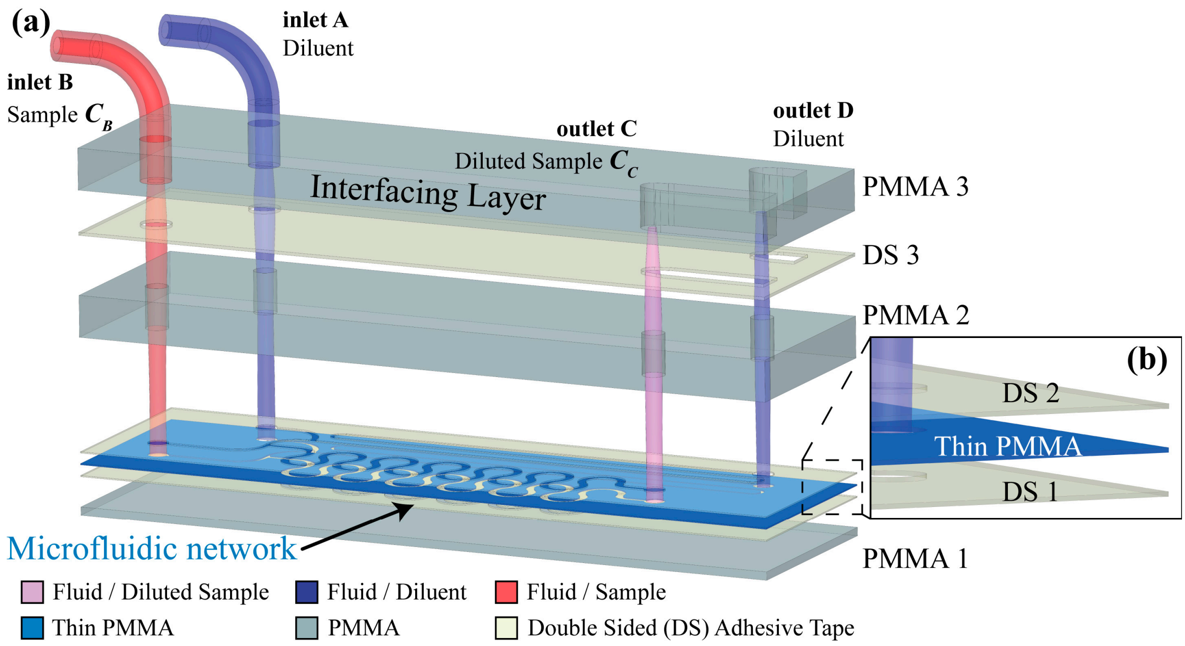

Figure 2S: Exploded view presenting the various PMMA and double sided adhesive tape (DS) layers used to fabricate the microfluidic PMMA prototype.

© 2019 by the authors. Submitted for possible open access publication under the terms and conditions of the Creative Commons Attribution (CC BY) license (http://creativecommons.org/licenses/by/4.0/).
